# The exploration of using plasma biomarkers of p-tau217 and p-tau181 for screening Alzheimer’s disease in very elderly people

**DOI:** 10.3389/fneur.2025.1668512

**Published:** 2025-10-16

**Authors:** Shouzi Zhang, Haiyan Wu, Li Ma, Rui Li, Li Zhang, Lixin Liu, Xuelin He, Tingyu Zhao

**Affiliations:** ^1^Department of Psychiatry, Beijing Geriatric Hospital, Beijing, China; ^2^State Key Laboratory of Cognitive Science and Mental Health, Institute of Psychology, Chinese Academy of Sciences, Beijing, China; ^3^Department of Psychology, University of Chinese Academy of Sciences, Beijing, China

**Keywords:** blood-based biomarkers, single molecule array (Simoa), p-tau181, p-tau217, GFAP

## Abstract

**Introduction:**

Blood-based biomarkers for Alzheimer’s disease (AD), such as phosphorylated tau (p-tau181, p-tau217) and amyloid beta (Aβ), have the potential to serve as screening tools for probable AD in the elderly population.

**Methods:**

AD screening [Mini-Mental State Examination (MMSE) and Montreal Cognitive Assessment (MoCA)] was conducted among very elderly individuals residing in a nursing community and a geriatric hospital. Based on cognitive evaluation, participants were categorized into two groups: cognitively normal (*n* = 62) and probable AD (*n* = 78). Plasma concentrations of Aβ42, Aβ40, p-tau181, p-tau217, and glial fibrillary acidic protein (GFAP) were measured using the single molecule array (Simoa) platform. Group comparisons of plasma biomarker levels were performed, and receiver operating characteristic (ROC) curve analyses were conducted for each biomarker relative to AD diagnosis.

**Results:**

Significant differences were observed in plasma p-tau181, p-tau217, and GFAP levels between the cognitively normal and probable AD groups (*p* < 0.01). In contrast, Aβ42, Aβ40, and the Aβ42/Aβ40 ratio showed no significant differences (*p* > 0.01). The area under the ROC curve (AUC) was 0.886 for p-tau181, 0.655 for p-tau217, and 0.869 for GFAP.

**Discussion:**

Plasma biomarkers p-tau181, p-tau217, and GFAP demonstrate clinical utility in distinguishing AD from normal cognition, suggesting that blood-based testing may serve as a feasible screening tool for early identification of AD in very elderly populations.

## Introduction

With the rising incidence of Alzheimer’s disease (AD) in China, early screening and diagnosis have become increasingly important. Currently, cerebrospinal fluid (CSF) biomarkers and amyloid positron emission tomography (PET) imaging are not widely applied in clinical practice due to their invasiveness, limited accessibility, and high costs. By contrast, blood-based biomarkers offer a more practical approach for screening and diagnosing AD, particularly in the context of disease-modifying therapies (DMTs) such as anti-amyloid immunotherapies, which primarily target individuals with mild cognitive impairment (MCI) or early-stage AD. Compared with CSF or PET examinations, plasma biomarkers are less invasive, more cost-effective, and easier to implement at the community level. As DMTs continue to develop, blood testing could serve as an efficient preliminary tool to identify patients with underlying AD pathology and to determine who should subsequently undergo confirmatory CSF or PET testing before initiating therapy.

Recent studies have demonstrated the diagnostic potential of several plasma analytes. For example, plasma Aβ42/Aβ40 correlates well with CSF biomarkers and amyloid PET, while plasma glial fibrillary acidic protein (GFAP) has been associated with increased dementia risk and accelerated cognitive decline. Furthermore, phosphorylated tau species, particularly p-tau181 and p-tau217, have been proposed as promising prescreening biomarkers, as they are elevated in Aβ-positive compared with Aβ-negative individuals (1). Both p-tau181 and p-tau217 exhibit strong diagnostic performance in distinguishing AD from cognitively normal individuals and from other forms of dementia. In older cohorts, these markers may help identify individuals with underlying amyloid and tau pathology (2). Pathologically, phosphorylated tau and Aβ are key components of neurofibrillary tangles and amyloid plaques, respectively. Plasma p-tau181 levels are reported to be 1.5–3.5 times higher in AD compared with cognitively normal controls, and 1.8–3.7 times higher in AD compared with clinically and pathologically confirmed frontotemporal lobar degeneration (FTLD) (3–5). CSF p-tau217, in particular, has demonstrated superior accuracy in differentiating AD dementia from other neurodegenerative diseases. When measured using the same immunoassay, CSF p-tau217 showed stronger correlations with amyloid- and tau-PET signals than p-tau181 (6). Similarly, large cohort studies have shown that plasma p-tau217 performs comparably to CSF p-tau217 and tau-PET, and outperforms plasma p-tau181 in clinical AD diagnosis (7).

However, very elderly individuals are often less willing to undergo invasive CSF testing or expensive PET imaging, due to factors such as frailty, comorbidities, and limited accessibility. To address this, we employed the commercially available Quanterix HD-X single molecule array (Simoa) platform, which measures plasma Aβ42, Aβ40, GFAP, p-tau181, and p-tau217. In a sample of very elderly individuals residing in nursing communities and geriatric hospitals, we combined plasma biomarker measurements with cognitive assessments to evaluate whether blood-based biomarkers retain diagnostic value for AD in this age group. The aim of this study was to investigate whether plasma biomarkers, including p-tau181, p-tau217, Aβ42, Aβ40, and GFAP, can serve as diagnostic tools for distinguishing AD from normal cognition in very elderly individuals.

## Methods

### Participants

Data were collected between January and May 2025 from a geriatric hospital and a nursing community. The cohort included cognitively normal controls (*n* = 62; mean age = 86.0 ± 5.72 years) and patients with clinically diagnosed AD (*n* = 78; mean age = 85.0 ± 5.35 years). AD diagnosis was made based on recommendations from the National Institute on Aging-Alzheimer’s Association workgroups on diagnostic guidelines for Alzheimer’s disease (8). All the patients had completed neuroimaging of structural MRI detection, and a small number of patients underwent cerebrospinal fluid biomarker testing or amyloid-β PET detection.

All AD participants presented with mild, moderate, or severe cognitive impairment, with Mini-Mental State Examination (MMSE) scores ranging from 0 to 23. None of the participants had a history of severe disease other than AD. The cognitively normal control group (*n* = 62) was defined by MMSE scores ≥ 27. There were no significant differences in age or sex distribution between the two groups.

Inclusion criteria required completion of both plasma biomarker measurements (Aβ42, Aβ40, Aβ42/Aβ40, p-tau217, p-tau181, and GFAP) and cognitive assessments (MMSE and MoCA). All participants provided written informed consent at the time of recruitment. The study protocol was approved by the Ethics Committee of Beijing Geriatric Hospital.

### Procedures

Blood samples were collected in the clinic after an overnight fast, centrifuged, aliquoted, and stored at −80 °C until analysis. Plasma biomarker measurements were performed at the Beijing Kingmed Clinical Laboratory using the single molecule array (Simoa) platform (Quanterix Corporation). The principles and technology of Simoa have been previously described (9). Additional details of the Neurology 4-Plex E assay for Aβ and tau quantification are available on the manufacturer’s website.^1^ Plasma Aβ40, Aβ42, and GFAP levels were measured using the Simoa Neurology 4-Plex E Advantage Kit (N4PE, item #103670). Plasma p-tau181 concentrations were measured on the HD-X Analyzer (Quanterix Corporation) with the Simoa p-tau181 Advantage Kit, version 2, following the manufacturer’s instructions. Plasma p-tau217 concentrations were measured on the HD-X Analyzer (Quanterix Corporation) using the Simoa pTau-217 Advantage Kit (Quanterix Corporation) according to the manufacturer’s instructions. All assays were performed by a board-certified laboratory technician using a single batch of reagents to minimize variability.

### Statistical analysis

Data were expressed as mean ± standard deviation (SD) or as absolute numbers. Group comparisons of clinical and demographic characteristics, cognitive assessments, and plasma biomarkers (AD vs. NC) were conducted using SPSS software. Between-group differences in plasma biomarkers were evaluated to control for potential confounding effects, using an ANCOVA, with age, sex, and MoCA entered as covariates. Spearman correlation analyses were performed to examine the associations between plasma p-tau181, p-tau217, and GFAP levels and MoCA scores among AD participants. The diagnostic performance of plasma biomarkers in discriminating AD from NC was assessed using receiver operating characteristic (ROC) curve analysis, with sensitivity and specificity calculated from the area under the curve (AUC). A two-tailed *p* < 0.05 was considered statistically significant.

## Result

A total of 140 participants were analyzed. No significant correlations between age or sex and plasma biomarker levels were detected in either group. Clinical and demographic characteristics, along with plasma biomarker data, are summarized in Table 1.

**Table 1:** Participants’ characteristics and plasma biomarkers assessed by Simoa platforms.

	NC (*n* = 62)	AD (*n* = 78)	*t*-value	*p*-value
Age, years	86 ± 5.72	85 ± 5.35		0.341
Sex, male	35	43		
MoCA	26 ± 1.76	6 ± 3.58	−8.209	0.000^**^
P-tau181	2.92 ± 1.74	5.62 ± 2.13	−8.171	0.000^**^
P-tau217	5.63 ± 8.25	15.72 ± 23.70	−3.356	0.001^**^
GFAP	24.30 ± 16.13	67.92 ± 61.76	−5.588	0.000^**^
Aβ42	51.59 ± 44.65	83.37 ± 110.92	−1.911	0.061
Aβ40	300.37 ± 214.72	339.55 ± 207.14	−0.890	0.378
Aβ42/Aβ40	0.36 ± 0.78	0.79 ± 2.94	−1.058	0.294

^**^p < 0.01 and ^*^p < 0.05.

**Figure 1:**
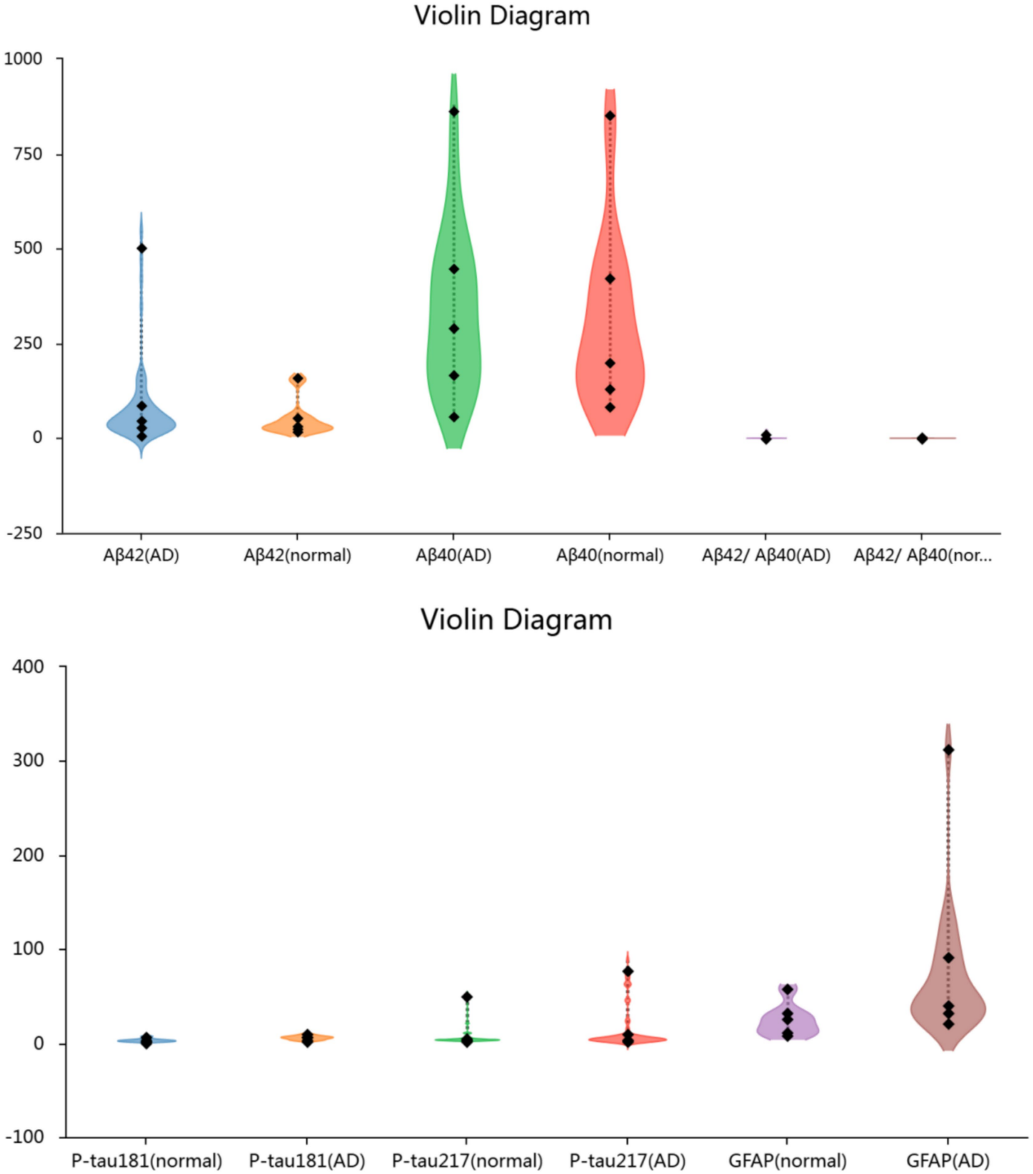
Plasma biomarkers levels in the two groups of participants.

Plasma levels of p-tau181, p-tau217, and GFAP were significantly higher in AD patients compared with NC (*p* < 0.01). In contrast, no significant differences were observed in plasma Aβ42, Aβ40, or the Aβ42/Aβ40 ratio between groups (Figure 1).

Correlation analyses demonstrated negative associations between MoCA scores and plasma biomarker levels of p-tau181 (*p* < 0.001), p-tau217 (*p* = 0.024), and GFAP (*p* = 0.002) among AD patients (Table 2).

**Table 2:** The Pearson’s relationship between plasma biomarkers and MoCA scores.

		MoCA
P-tau181	Pearson’s *R*	−0.482
	*p*-value	0.000^**^
P-tau217	Pearson’s *R*	−0.226
	*p*-value	0.024^*^
GFAP	Pearson’s *R*	−0.303
	*p*-value	0.002^**^

^**^p < 0.01 and ^*^p < 0.05.

**Figure 2:**
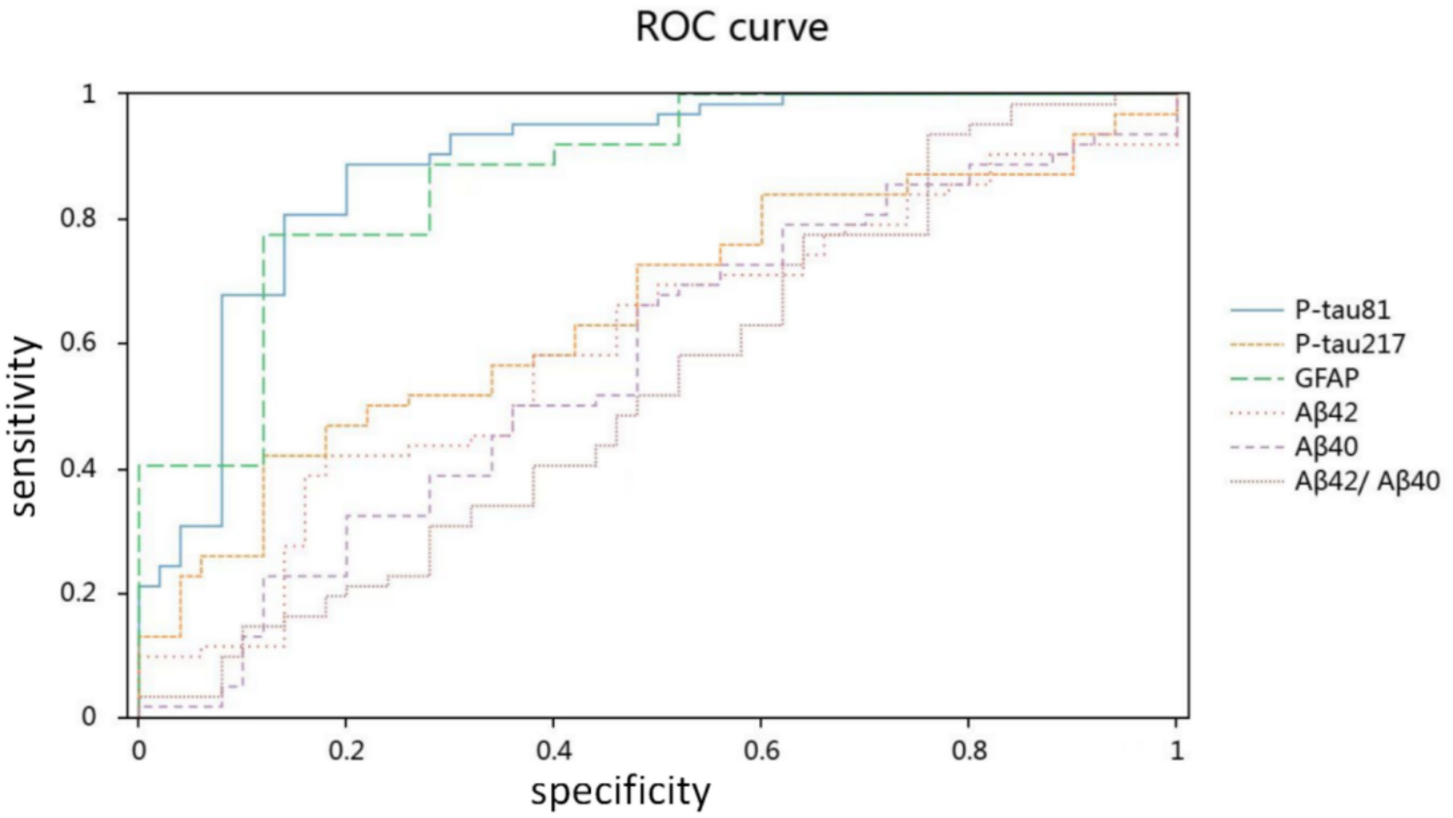
Diagnostic accuracy of plasma biomarkers for Alzheimer’s disease.

The ROC analysis revealed that the strongest diagnostic performance was achieved by p-tau181 (AUC = 0.886, 95% CI: 0.821–0.951) and GFAP (AUC = 0.869, 95% CI: 0.803–0.935). In contrast, p-tau217 (AUC = 0.655, 95% CI: 0.555–0.756) and Aβ42 (AUC = 0.605, 95% CI: 0.499–0.710) demonstrated lower diagnostic accuracy. The remaining biomarkers showed AUCs ranging from 0.548 for the Aβ42/Aβ40 ratio (95% CI: 0.438–0.658) to 0.578 for Aβ40 (95% CI, 0.470–0.685) (Figure 2 and Table 3).

**Table 3:** AUC for plasma biomarkers in ROC.

	AUC	*p*	95% CI
P-tau181	0.886	0.000^**^	0.821–0.951
P-tau217	0.655	0.005^**^	0.555–0.756
GFAP	0.869	0.000^**^	0.803–0.935
Aβ42	0.605	0.058	0.499–0.710
Aβ40	0.578	0.158	0.470–0.685
Aβ42/Aβ40	0.548	0.385	0.438–0.658

^**^p < 0.01 and ^*^p < 0.05.

## Discussion

In 2024 the Alzheimer’s Association Workgroup revised the diagnostic and staging criteria for AD. The updated framework highlights core 1 biomarkers, including CSF or plasma Aβ42, p-tau217, p-tau181, p-tau231, and amyloid PET, all of which map onto either the amyloid beta pathway or the AD tauopathy pathway (10). Core 2 biomarkers include tau proteinopathy markers such as microtubule-binding region tau (MTBR-tau243) and p-tau205, which correlate more strongly with tau PET than with amyloid PET (11, 12). The revised framework also emphasizes other biomarkers that reflect non-specific processes in AD pathophysiology. These include CSF or plasma neurofilament light chain (NfL) and GFAP, which indicate axonal injury, neuronal dysfunction, synaptic degeneration, and neuroinflammation. In addition, the new criteria introduced biomarker categories for vascular brain injury (V) and alpha-synucleinopathy (S). These categories are relevant to AD diagnosis and staging because AD frequently co-occurs with other pathologies and comorbid processes play an important role in disease progression (10). We refer to this framework in order to situate our findings within the most recent international consensus on biomarker-based diagnosis, rather than to propose a novel classification. The biomarker-positive cognitively normal individuals should be considered as being at risk for AD, but cannot be diagnosed with AD according to recommendations of the International Working Group (13). These new guides highlight the diagnostic value of plasma biomarkers, demonstrating their growing recognition in clinical practice, which also underscores the value of our research.

Our results confirmed that tau-related plasma biomarkers were altered in very elderly AD patients, in line with previous studies (14–16). Among the tau isoforms, p-tau181 and p-tau217 have consistently been highlighted as strong diagnostic markers in both CSF and plasma (7, 17). Brickman et al. (16) measured plasma biomarkers in 113 autopsied participants, 29% of whom had high AD neuropathological changes, and in 300 clinically evaluated individuals, 42% of whom were diagnosed with AD. Their results indicated that plasma p-tau181, p-tau217, and NfL concentrations were elevated in both pathologically and clinically diagnosed AD. In addition, a decreased Aβ42/Aβ40 ratio together with increased p-tau217 and p-tau181 was associated with subsequent AD diagnosis (16). In our cohort of very elderly participants, plasma p-tau181 and GFAP demonstrated stronger diagnostic performance than p-tau217. This finding contrasts with previous reports where p-tau217 outperformed other plasma markers, particularly in younger or mixed-age cohorts. Several factors may explain this discrepancy. First, the relatively small sample size in our study may have limited statistical power. Second, very elderly individuals often present with multiple comorbid pathologies, such as vascular brain injury and neuroinflammation, which may alter biomarker dynamics and attenuate the discriminative value of p-tau217. Janelidze et al. (4) have established the high diagnostic accuracy of p-tau217 but also noted variations in performance across cohorts, which could be influenced by age and comorbid factors. A higher prevalence of co-pathologies (vascular lesions or TDP-43 pathology) and altered blood–brain barrier dynamics might differentially affect the sequestration, clearance, or expression of these p-tau isoforms in biofluids, thereby modulating their diagnostic performance. Third, technical factors including assay sensitivity and batch effects may also have contributed. These considerations suggest that our findings should be interpreted with caution, and further validation in larger longitudinal cohorts is warranted.

Previous studies in younger or mixed-age cohorts consistently reported that plasma p-tau217 outperformed p-tau181 and amyloid-related markers in detecting early AD pathology (6, 7, 16, 18). In contrast, our results suggest that in a very elderly cohort, p-tau181 and GFAP may provide stronger diagnostic accuracy. Importantly, several independent studies have also supported the robustness of p-tau181 and GFAP as biomarkers in clinical and elderly populations. Baiardi et al. (19) demonstrated that both plasma p-tau181 and GFAP show strong diagnostic value in dementia screening cohorts. Ingannato et al. (20) further reported that GFAP and p-tau181 levels increase across SCD, MCI, and AD, highlighting their utility in prodromal and symptomatic stages. GFAP, in particular, has been validated as an astrocytic marker closely associated with amyloid pathology (21), and has shown stability in longitudinal tracking of AD-related decline (22). Moreover, a recent community-based study in older adults found that elevated baseline p-tau181 and GFAP—but not p-tau217—were more strongly associated with future dementia onset in individuals aged 78 years and above (23).

Although plasma p-tau217 has been widely validated and recently approved by the FDA for clinical use, our findings suggest that its discriminative accuracy may be attenuated in very elderly populations compared with p-tau181 and GFAP. On May 16, 2025, the U.S. Food and Drug Administration (FDA) approved the first *in vitro* diagnostic device, the Lumipulse G pTau217/β-Amyloid 1–42 Plasma Ratio, for clinical use to aid in the diagnosis of AD (24). The FDA evaluation was based on data from a multicenter clinical study involving 499 plasma samples collected from cognitively impaired adults. Plasma test results using the Lumipulse G pTau217/β-amyloid 1-42 plasma ratio were compared with amyloid PET scans and CSF biomarkers. In this study, 91.7% of individuals with positive plasma results had evidence of amyloid plaques confirmed by PET or CSF, whereas 97.3% of those with negative plasma results were amyloid-negative by PET or CSF. Plasma phosphorylated tau 217 (p-tau217) has been shown to be one of the most accurate diagnostic markers for AD. Both Lumipulse and single molecule array (Simoa) technologies are widely used to measure plasma p-tau217 and p-tau181 levels. In a head-to-head comparison between Lumipulse and ALZpath Simoa assays, plasma p-tau217 demonstrated excellent diagnostic accuracy for detecting AD. The two platforms showed comparable performance in distinguishing AD from other neurodegenerative diseases (18).

Simoa technology was applied to measure plasma biomarkers in our study. In recent years, low-sensitivity plasma assays have been replaced by ultra-sensitive platforms such as Mesoscale Discovery (MSD), Simoa, and immunoprecipitation-mass spectrometry (IP-MS), all of which offer improved accuracy in detecting plasma biomarkers of AD (6, 25–27). Further validation is still required to determine which of these methods can be most effectively promoted for clinical use. Electrochemiluminescence (ECL) immunoassay and Simoa currently represent the most promising approaches, and the development of accurate and efficient reagent kits will also be critical for precise biomarker detection. Beyond Aβ42 or Aβ40, the diagnostic accuracy of plasma p-tau217 for detecting Aβ pathology has been demonstrated in several studies (28–31). However, our findings indicated that plasma p-tau181 and GFAP were more accurate than p-tau217 in distinguishing cognitive impairment in the very elderly population. This discrepancy may reflect the relatively small sample size in our study, or it may indicate that AD patients in advanced age groups present different biomarker profiles compared with younger populations. Taken together, our results suggest that plasma p-tau217, p-tau181, and GFAP have diagnostic value in distinguishing AD from normal cognition in the very elderly population. The cross-sectional design does not allow conclusions regarding predictive value, and further longitudinal studies are needed.

The difference in plasma Aβ levels between the patient group and the control group did not reach statistical significance. A part of AD patients included in this study had confirmed β-amyloid pathology via CSF or PET imaging. This lack of significant difference may be attributed to the limited sensitivity of the Aβ assay kit used, which could have impeded the accurate detection of trace amounts of Aβin plasma. However, the possibility cannot be ruled out that some patients clinically diagnosed with AD may actually have other underlying neuropathologies, such as primary age-related tauopathy (PART) which is an Aβ-independent accumulation of neurofibrillary degeneration in medial temporal lobe structures. The cognitive status in PART subjects has been shown to be related to the overall hippocampal p-tau burden, as well as the presence of additional comorbid neuropathologic findings (32).

Limitations of this study should be acknowledged. First, the sample size was relatively small, which may have limited statistical power to detect more subtle biomarker effects. Second, plasma biomarkers were not validated against CSF or PET measures in this very elderly cohort. Most participants were unwilling or unable to undergo lumbar puncture or amyloid/tau PET imaging due to frailty, comorbidities, and limited accessibility, so diagnosis was based on clinical criteria according to the NIA–AA 2011 guidelines rather than biomarker confirmation. This limits the strength of claims regarding diagnostic reliability. Third, normative reference values and cut-off points for plasma biomarkers were not established, which constrains clinical applicability. Taken together, these limitations underscore the need for replication in larger, longitudinal, and multimodal studies with CSF or PET validation and standardized thresholds to confirm the diagnostic accuracy of plasma biomarkers in very elderly populations.

## Data Availability

The original contributions presented in the study are included in the article/supplementary material, further inquiries can be directed to the corresponding authors.

## Footnotes

^1^ www.quanterix.com
